# Institutional Needs

**Published:** 1915-11-06

**Authors:** 


### INSTITUTIONAL NEEDS.

### "AUSTRAL" WINDOWS.

(THE "AUSTRAL" WINDOW BALANCE CO., LTD., 3 Macdonald's Lane, Corporation Street, Manchester.)

A WINDOW that is cheap in construction, simple in mechanism, and effective in providing means for the admission of fresh air into hospitals—temporary or permanent—is worthy of notice. Such a window is furnished by the "Austral" Window Balance Co., Ltd., who have submitted to us two booklets containing full details as to the construction of the windows and many illustrations. It has been the endeavour of the inventors to produce a window which shall provide for a fresh-air inlet in an upward direction, the maximum of light with fewest obstructions, no receptacles for harbouring dust, and the regulated admission of air in all weathers without draught. It is claimed that the "Austral" window contains all these and other advantages, and that it has been declared by many architects to be "the hospital window of the future." Hospital authorities would be well advised to obtain full particulars from the makers and to inquire closely into the relative merits of this window.

